# *Streptococcus ruminantium*-associated sheep mastitis outbreak detected in Italy is distinct from bovine isolates

**DOI:** 10.1186/s13567-023-01248-9

**Published:** 2023-12-12

**Authors:** Maria Nives Rosa, Ben Vezina, Gavino Marogna, Antonella Canu, Monica Rosaria Molotzu, Sebastiana Tola

**Affiliations:** 1 Istituto Zooprofilattico Sperimentale della Sardegna “G. Pegreffi”, 07100 Sassari, Italy; 2 Department of Infectious Diseases, Central Clinical School, Monash University and the Alfred Hospital, Melbourne, VIC Australia

**Keywords:** Sheep, ovine, mastitis, outbreak, *Streptococcus ruminantium*, antimicrobial susceptibility, whole-genome sequencing

## Abstract

*Streptococcus ruminantium* is the causative agent of several bovine and ovine diseases, however reports are uncommon and application of whole genome sequencing to identify is rare. We report for the first time, a severe ovine mastitis outbreak caused by *S. ruminantium* in Italy, 2022. *S. ruminantium* was isolated from 12 adult lactating ewes with diffuse nodules in the mammary parenchyma and predominantly serous and clotted milk. All outbreak isolates, along with five additional historical Italian isolates (between 2011 and 2017), were genomically characterised and then analysed in the context of all publicly available *S. ruminantium* genomes. Antimicrobial susceptibility testing was performed to determine the MICs of 16 antibiotics. The results showed that all isolates were susceptible to all antimicrobials tested except kanamycin. Single Nucleotide Variant analysis confirmed this as a clonal outbreak across 10 sheep (≤ 15 SNVs), while the two others were colonised by more distantly related clones (≤ 53 pairwise SNVs), indicating the presence of multiple infecting lineages. The five historical *S. ruminantium* isolates were comprised of genetically-distant singletons (between 1259 and 5430 pairwise SNVs to 2022 outbreak isolates). Ovine isolates were found to be genetically distinct to bovine isolates, forming monophyletic groups. Bovine isolates were similarly made up of singleton clones in all but two isolates. Taken together, our genomic analysis using all globally available genomes is consistent with general opportunistic pathogenesis of *S. ruminantium*. We encourage future genomic surveillance efforts to facilitate outbreak detection, as well as improve our understanding of this poorly-understood, multi-host, zoonotic pathogen.

**Supplementary Information:**

The online version contains supplementary material available at 10.1186/s13567-023-01248-9.

## Introduction

Mastitis is one of the most common and costly diseases of dairy sheep and goats. Infectious mastitis outbreaks can be caused by a variety of bacterial species, including the genera *Staphylococcus* and *Streptococcus* [1–3]. In both genera, new species are constantly being discovered or renamed through the application of genomic analysis. *Streptococcus* (*S*.) *ruminantium* was first proposed as a name for a novel species in 2017 by Tohya et al. [4], based on phylogenetic analysis of 16S *r*RNA and *sod*A gene sequences. *S. ruminantium* was previously classified as serotype 33 of *S. suis*, one of the most important swine pathogens responsible for various diseases including pneumonia, arthritis, meningitis and endocarditis [5]. It is also a zoonotic agent that affects humans in close contact with infected pigs or pig products [6]. As it is difficult to differentiate *S. suis* from *S. ruminantium* in routine diagnostics based on the use of biochemical tests, either whole genome sequencing or specific PCRs can be used for differential identification [7–11].

Following the classification of *S. ruminantium* as a new species, five case reports associated with this species in ruminants have been published along with 24 publicly available genomes. Okura et al. [11] reported the isolation of *S. ruminantium* from cattle with endocarditis, arthritis and pneumonia or respiratory disease in Japan, while Gottschalk et al. [12] described the bacterial isolation from 10 cattle and 4 sheep with arthritis, pneumonia, endocarditis and abscesses in Canada. Both reports lacked information on clinical signs and in some cases have not made sequence data publicly available. Recently, *S. ruminantium* was associated with pathology in 4 Pyrenean chamois and 1 domestic sheep from NE Spain [13]. In wild ruminants, *S. ruminantium* has been isolated from lung lesions and heart valve vegetations, and in domestic ruminants from liver abscesses [13].

The present study provides the first information on the association of *S. ruminantium* with an outbreak of mastitis in dairy ewes in Sardinia, Italy. We describe the clinical signs of the animals, the isolation and identification of the bacterial species associated with mastitis. All *S. ruminantium* isolates were subjected to molecular and genome sequence analysis to gain insight into bacterial clonality, pathogenicity and the presence of antimicrobial resistance genes.

## Materials and methods

### Farm and clinical examination

The owner of the dairy sheep farm, located in the eastern part of Sardinia, contacted the Istituto Zooprofilattico Sperimentale della Sardegna (IZSSA) in 2022 due to several cases of clinical mastitis. Following contacts with the farmer and the farm veterinarian, an outbreak investigation was initiated to detect and identify the microorganism, a possible source of the organism and to control the outbreak.

During the farm visit (31/05/2022), information was obtained from the farmer on farm type, flock size, milking and hygiene practices including use of disinfectants and antibiotics, previous vaccinations, and type of milking (manual or mechanical). Clinical examination was performed on each half-udder and included a general inspection, assessment of half-udder consistency, macroscopic examination of milk and palpation of supramammary lymph nodes. During the examination, the presence of pustules, crusts, corneal growths, ulcers, nodules, abscesses, *rubor*, *calor* and *dolor* (by warmth on palpation) was assessed. The consistency of the udder on palpation was classified as normal, edematous, sclerotic, or atrophic. The macroscopic appearance of the milk was classified as: normal, serous, haemorrhagic, presence of clots or absence of secretion [14].

Out of the 174 lactating ewes, 24 were found to have clinical mastitis. Twelve ewes were selected based on common udder clinical pictures, and milk and blood samples were taken from these ewes.

### Milk sampling and microbiological cultures

From each of the 12 selected ewes, we collected at least 5 ml of milk from both half-udders in a sterile container, after cleaning the teat with 90% denatured ethanol and discarding the first drops of milk in a bucket containing sodium hypochlorite. Samples were refrigerated and subjected to microbiological examination in the laboratory within 12 h from collection. For microbiological cultures, 10 μL of milk were seeded in 5% sheep blood agar and incubated at 37 ± 1 °C for 24–48 h. Only pure bacterial colonies grew in the 12 blood agar plates. All isolates were identified at the genus level based on growth characteristics, morphology of colonies and microscopic examination after Gram staining.

### MALDI-TOF MS identification

In this study, we analysed all 12 outbreak isolates, two *S. suis* isolates (3089 and 3627) collected from pigs with pneumonia, and five historical isolates (OM1195, OM2067, OM2267, OM2774 and OM3442) belonging to our bank and collected from sheep mastitis in previous years (Additional file 2). These historical isolates, archived as *S. suis*, were re-identified to understand whether the circulation of *S. ruminantium* in Sardinia was before 2022. For MALDI-TOF MS analysis, the direct colony transfer protocol was applied. Each target plate included two spots of Bacterial Test Standard (Bruker Daltonik GmbH). Mass spectra were acquired using the MALDI-TOF MS spectrometer in a linear positive mode (MALDI Biotyper Sirius One, Bruker Daltonics). The MBT Compass library revision K (2022), covering 4274 species/entries, were used for bacterial identification (Additional file 1). The similarity of patterns was represented as a score, according to manufacturer specifications: a score value of < 1.7 indicated that identification was not reliable; scores between 1.7–2.0 that identification was reliable at the genus level; scores between 2.0 and 2.3 that identification was reliable at the genus level and probable at the species level; scores higher than 2.3 indicated highly probable species identification.

### DNA extraction, polymerase chain reaction (PCR) amplification, and restriction fragment length polymorphism (RFLP) analysis

Genomic DNA was extracted from the 19 (12 outbreak, 2 *S. suis* and 5 historical) isolates as described by Onni et al. [15], while species identification was based on PCR amplification of the glyceraldehyde-3-phosphate dehydrogenase gene (*gap*) gene (Table 1). Fifteen microliters of both PCR amplifications were digested in a 30 µL volume containing 10 × FastDigest Green buffer, 0.25 µL of 20 mg/mL acetylated BSA, and 1 µL of FastDigest AluI enzyme (Thermo Scientific, CA, USA). Reaction mixtures were incubated at 37 °C for 15 min and directly loaded on the precast NuPAGE™ gels (Invitrogen).

**Table 1:** **Oligonucleotide sequences used in this study.**

Target gene	Primer name	Primer sequence (5ʹ—3ʹ)	T_A_ (°C)	Amplicon size (bp)	References
*recN*	SSrecN-F SSrecN-R	CTACAAACAGCTCTCTTCT ACAACAGCCAATTCATGGCGTGATT	60	336	[7]
*gdh*	JP4 JP5	GCAGCGTATTCTGTCAAACG CCATGGACAGATAAAGATGG	55	688	[10]
*16S rRNA*	F R	GCAAGTGGAACGCAACTTTTCA CTATGTATCGTTGCCTTGGTAG	60	240	[11]
*gap*	Strept-gap-F Strept-gap-R	ACTCAAGTGTACGAACAAGT GTCTTGCATTCCGTCGTAT	54	945	[16]

### Amplicon sequencing, PCR for *S. ruminantium* and pulsed field gel electrophoresis (PFGE)

The *gap* gene amplicons of all 19 isolates were sequenced at BMR Genomics with the Sanger sequencing option. The nucleotide sequences were compared to sequences in the GenBank database using the Basic Local Alignment Search Tool (BLAST).

The primers used for *S. ruminantium* identification are listed in Table 1. After PCR reactions, amplicons were analyzed by electrophoresis on agarose gels.

Isolates were genotyped by PFGE. DNA was extracted with Bio-Rad CHEF genomic DNA plug kit (BioRad, Segrate, Italy) according to the manufacturer’s instructions. Each plug was digested with 20 U of SmaI (Roche) for 3 h at 25 °C. Electrophoresis was carried out in a contour-clamped homogeneous electric field (CHEF)- Mapper system (BioRad) at 14 °C in 0.5 × TBE buffer. DNA fragments were separated after 18 h migration with 6 V/cm, 120° at pulse times of 10–45 s. The digitalized PFGE patterns were analyzed with the Gel Compar II software (Applied Maths, Sint-Martens-Latem, Belgium).

### Antimicrobial susceptibility testing

The Minimum Inhibitory Concentration (MIC) of 16 antimicrobial agents were determined by the broth microdilution method using the Sensititre^™^ ITISVE8 plates (Thermo Fisher Scientific, West Sussex, UK). The customized plates were prepared for Istituto Zooprofilattico della Lombardia e Emilia Romagna to test the antimicrobial susceptibility of Gram-positive bacteria responsible of ovine and caprine mastitis. The antimicrobials tested were: amoxicillin/clavulanic acid 2:1 ratio (AUG2, 16/8–0.25/0.12 µg/mL), enrofloxacin (ENRO, 4–0.25 µg/mL), cefazolin (FAZ, 8–0.25 µg/mL), ceftiofur (XNL, 8–0.25 µg/mL), erythromycin (ERY, 8–0.03 µg/mL), florfenicol (FFN, 8–2 µg/mL), tetracycline (TET, 16–0.25 µg/mL), kanamycin high level (KAN HL, 500–250 µg/mL), kanamycin (KAN, 32–8 µg/mL), trimethoprim/sulfadiazine (TBR, 8/152–0.12/2.38 µg/mL), oxacillin + 2% NaCl (OXA + , 4–0.25 µg/mL), rifampin (RIF, 2–0.06 µg/mL), clindamycin (CLI, 2–0.5 µg/mL), sulfisoxazole (FIS, 512–128 µg/mL), ampicillin (AMP, 16–0.03 µg/mL), tilmicosin (TIL, 32–8 µg/mL), and penicillin (PEN, 16–0.03 µg/mL). The MIC test conditions were performed according to the manufacturer’s instructions. Plates were incubated at 37 °C for 24 h and the reading was performed manually. Where possible, results were interpreted according to CLSI breakpoints from other animals or other *Streptococcus* species [17], otherwise the EUCAST range was used [18]. All 17 (12 outbreak and 5 historical) *S. ruminantium* isolates used in this study, antimicrobials tested and their breakpoints are listed in Additional file 2.

### Whole-genome sequencing

Genomes from all 17 *S. ruminantium* isolates were prepared using the enzymatic lysis method as described previously [19]. Sequencing libraries were made using Ion Xpress™ Plus Fragment Library Kit (Thermo Fisher Scientific) according to the manufacturer’s instructions. Libraries were sequenced with an IonTorrent Personal Genome Machine (PMG) (Life Technologies, Carlsbad, CA) at the IZSSA.

### Genome acquisition and assembly

For data generated in this study, SPAdes [20] version 3.15.3 was used to assemble the IonTorrent reads with the following options: “–iontorrent –isolate”. K-mers were iterated through starting with −k 27, then 53,71,87,99,111,119,127 with the “–restart-from k[53…127]”. After these 8 assemblies were complete, the.gfa files were evaluated using GFA-dead-end-counter version 1.0.0 [21]. Assemblies with the fewest Graphical Fragment Assembly (GFA) dead ends, then fewest contigs were chosen as the best assembly. Assemblies with > 20 GFA dead ends were re-sequenced.

For data obtained from other studies, sequencing reads were obtained from Sequence Read Archive where possible, or as assemblies from Genbank if raw reads were unavailable. In total, 3 readsets and 21 assemblies were obtained from three studies [11, 22, 23]. For genomes with long and short reads available, long reads were assembled using Flye version 2.8.1-b1676 [24] with the following options: “–nano-raw -g 2100000–asm-coverage 80 –plasmids”. Assemblies were long read polished using medaka version 1.8.0 with the following option: “medaka_consensus −m r941_min_high_g360”. Paired short reads were then trimmed with Trim Galore version 0.5.0 using the −q 20 option [25]. BWA-MEM version 0.7.17 [26] was used to align each paired short read file to the long read assembled genome, then Polypolish version 0.5.0 was used for short read polishing [27].

### Average nucleotide identity

To confirm the species identification, Average Nucleotide Identity (ANI) was calculated in a pairwise fashion between all isolates identified in this study and all known *S. ruminantium* genomes, along with *Streptococcus suis* (accession: GCF_000026745.1) using FastANI version 1.33 [28] with “–fragLen 1000”.

### Single nucleotide variant analysis

Single Nucleotide Variant (SNV) analysis was used to determine outbreak potential of isolates. This was performed using SKA version 1.0.0 [29] using the “ska fasta”, “ska distance -s 20” then “ska compare” options. SNV thresholds were determined by plotting a histogram of the pairwise SNVs between potentially linked isolates.

### Isolate clustering

PopPUNK version 2.4.0 [30] was used to assign genomes to clusters. The “create-db” function was used with the following options: “–sketch-size 1 000 000 –min-k 15 –max-k 29 –qc-filter prune”. Then the “fit-model” function was used with the following options: “bgmm -K 5 –ranks 1,2,3,5 –graph-weights”. Then the “fit-model” function was used with the following options: “refine –graph-weights –unconstrained”. Then the “poppunk_visualise” function was used, with the “—distances” and “–previous-clustering” utilising the refined model fit, to output a neighbour-joining core tree.

### Genome annotation

Bakta version 1.5.1 [31] was used to annotate all remaining genomes. Bakta database accessed on 18/08/2022).

### Antibiotic resistance screening

AMRFinderPlus version 3.10.23 [32] was used to screen assemblies for antibiotic resistance genes using protein input with identity set to 80%: “-i 0.8”.

### Pangenomics

The pangenome was constructed using Panaroo version 1.2.8 [33] with the following options: “–clean-mode sensitive -a core –aligner mafft –no_clean_edges –core_threshold 0.98 –merge_paralogs –remove-invalid-genes”.

### Putative virulence factor screen

To identify putative virulence factors within *S. ruminantium* genomes, a custom database was constructed using the abricate command “—setupdb”. Sequences were obtained using closely-related *Streptococcus suis* studies [34, 35], then duplicates removed. This is available as Additional file 3. Blastp (Blast + version 2.9.0 [36]) was used with the following options: blastp -outfmt “6 qseqid sseqid pident length qcovs” “-max_hsps 1 -num_alignments 1” then filtered on 80% query coverage and identity to identity positive hits.

### Visualisation

Visualisation was performed in R version 4.1.2 [37], RStudio version 1.4.1717 [38], with the following software packages: tidyverse version 1.3.1 [39], RColorBrewer version 1.1–2 [40], igraph version 1.2.7 [41], ggraph version 2.0.5 [42], aplot version 0.1.6 [43], ape version 5.5 [44], ggtree version 3.7.1.003 [45], cluster version 2.1.2 [46] ggforce version 0.3.3 [42]. All R code can be found in Additional file 4.

### Data availability

All assemblies and reads have been uploaded to Genbank under BioProject Accession PRJNA1009676. BioSample accessions can be found in Additional file 2.

## Results

### Flock and clinical examination

The Sardinian flock, consisting of 174 adult lactating ewes, was of the semi-wild type with the co-presence of other animal species such as horses and pigs but no cows. The ewes were grouped into a clean environment for milking only. The trolley milking machine was equipped with a single bucket, two teat cups, and a vacuum pump. The parameters set were: vacuum (−41 kPa), pulsation (180) and percentage between vacuum and atmospheric pressure at the teat (50%). The main palpable clinical signs in the 12 sheep with mastitis are shown in Table 2. The most common findings were enlarged supramammary lymph nodes and the presence of indicators of chronic infection such as nodules, sclerosis and atrophy of half or both mammary parenchyma. However, an overlap between chronic and acute infection was found in almost all animals, due to the simultaneous presence of clinical signs such as *rubor*, *calor*, *dolor*, edema and the presence of blood in the milk. Nine out of the 16 milk samples were serous with clots. No antimicrobial treatment was given to infected sheep.

**Table 2:** **Clinical examination of udder, lymph nodes, and milk of the 12 ewes with mastitis.**

Sheep n°	Half-udder RH	Half-udder LH	SLN RH	SLN LH	Milk
2627	Nodules, Calor, Rubor Sclerotic, Atrophic	Calor, Edematous	Reactive	Reactive	Normal
6843	Edematous	Sclerotic, Atrophic	Reactive	Normal	RH = slightly serous LH = serous/hemorrhagic
671	Edematous Sclerotic	Edematous	Reactive	Reactive	RH = serous with clots
8464	Nodules Edematous	Sclerotic, Atrophic	Normal	Normal	RH = serous with clots LH = serous with clots
8206	Sclerotic, Atrophic	Slightly edematous	Reactive	Normal	RH = serous with clots
2605	Nodules Edematous	Atrophic	Reactive	Normal	RH = hemorrhagic
8477	Dolor Sclerotic Atrophic	Edematous	Reactive	Reactive	RH = serous with clots
6740	Nodules Edematous Sclerotic	Nodules	Reactive	Reactive	RH = slightly serous with clots
6730	Nodules Edematous Sclerotic	Nodules	Reactive	Reactive	RH = serous with clots
1519	Nodules Calor Edematous	Nodules Calor Edematous	Reactive	Reactive	RH = serous LH = serous
2622	Calor Rubor Edematous	Edematous Sclerotic	Normal	Reactive	RH = serous with clots and slightly hemorrhagic LH = serous
2616	Nodules	Edematous	Reactive	Reactive	RH = serous with clots

RH:  Right

LH:  Left

SLN: Supramammary lymph nodes

### Molecular identification of *S. ruminantium*

Prior to whole genome sequencing, an attempt was made to identify the 12 outbreak isolates by MALDI-TOF MS, but the result was “No Organism Identification Possible”, with a score < 1.33. RFLP also showed distinct profiles of these 12 isolates compared to two field isolates (3089 and 3627) collected from pig lung lesions and identified as *S. suis* by MALDI-TOF MS with a score > 2.3 (Additional file 5). PCR amplification and sequencing of the *gap* gene showed 97% identity and 99% query coverage to *S. ruminantium* (accession: CP019557.1) (Additional file 6).

All the 12 *S. ruminantium* isolates and the two *S. suis* isolates were PCR positive for the *gdh* gene, whereas all *S. ruminantium* isolates were negative for *S. suis*-specific *rec*N-PCR, while positive for *S. ruminantium*-specific 16S *r*RNA-PCR (Additional file 7). The historical isolates showed the same characteristics (MALDI-TOF, RFLP and PCR) as the 12 outbreak *S. ruminantium* isolates.

Molecular identification of the 17 *S. ruminantium* was confirmed by whole-genome sequencing and average nucleotide identity (ANI), with all isolates falling within 98.3% ANI of 21 published *S. ruminantium* genomes.

### Outbreak analysis

**Figure 1:**
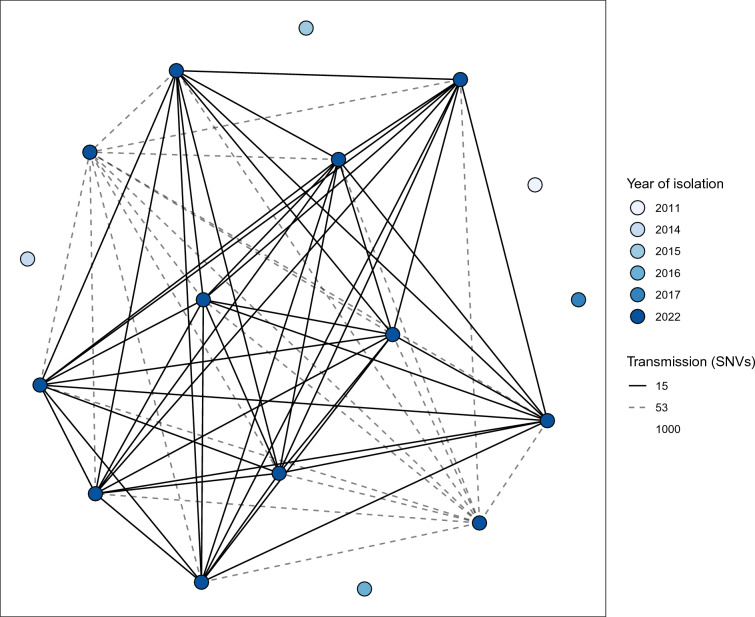
**Pairwise single nucleotide variant (SNV) network.** Isolates are shown as nodes, coloured by year of isolation. Transmission was set at ≤ 15 SNVs (solid lines), with more distantly related isolates found with ≤ 53 SNVs (dotted line). Isolates from previous years with ≥ 1000 pairwise SNVs have blank lines.

Initially, PFGE was performed to rapidly assess the degree of genetic diversity of the 12 *S. ruminantium* isolates, which indicated a high degree of genetic similarity and thus spread within the flock (Additional file 8). Pairwise SNV analysis after whole genome sequencing confirmed this finding at higher resolution. Comparison of all pairwise SNVs between the 12 Sardinian isolates suspected to be part of an outbreak showed three clear SNV boundaries, first at ≤ 13 SNVs, then between ≥ 30 to ≤ 53 SNVs, then > 53 SNVs (Additional files 9 and 10). Given the epidemiological context of this event, that all isolates were collected on a single day from the same Sardinian flock, and the distribution of pairwise SNVs, direct transmission was set at ≤ 15 SNVs. The SNV network provides strong evidence that a clonal outbreak cluster of 10 sheep occurred on this farm in 2022 (Figure 1). The presence of two more distantly related isolates (within 53 pairwise SNVs to the other 2022 isolates) indicated the presence of multiple infecting lineages or lineages that had recently diverged. This suggests that different *S. ruminantium* lineages may be more prevalent in sheep populations than previously thought. Historical isolates collected throughout Sardinia between 2011 and 2017 were genetically distant from the 2022 outbreak isolates (1259–5430 pairwise SNVs) (Additional file 10).

### Antimicrobial susceptibility and resistance gene determinants

All the 17 *S. ruminantium* isolates were susceptible to AUG2, FAZ, XNL, FFN, TBR, OXA + , RIF, CLI, FIS, AMP, TIL and PEN, as no growth was observed at the lowest concentration of antimicrobial tested. MIC values were determined for TET and ENRO, which can be considered as susceptible breakpoints according to the guidelines described in Materials and methods. Isolates grew at all the lowest concentrations of KAN tested (32-16-8 µg/mL) but not at the highest (500–250 µg/mL). (Additional file 2). Based on the AMRFinderPlus analysis (dated 31/07/2023), no antimicrobial resistance genes were found in the *S. ruminantium* isolates (Additional file 11).

### Cow and sheep isolates are genomically distinct

**Figure 2:**
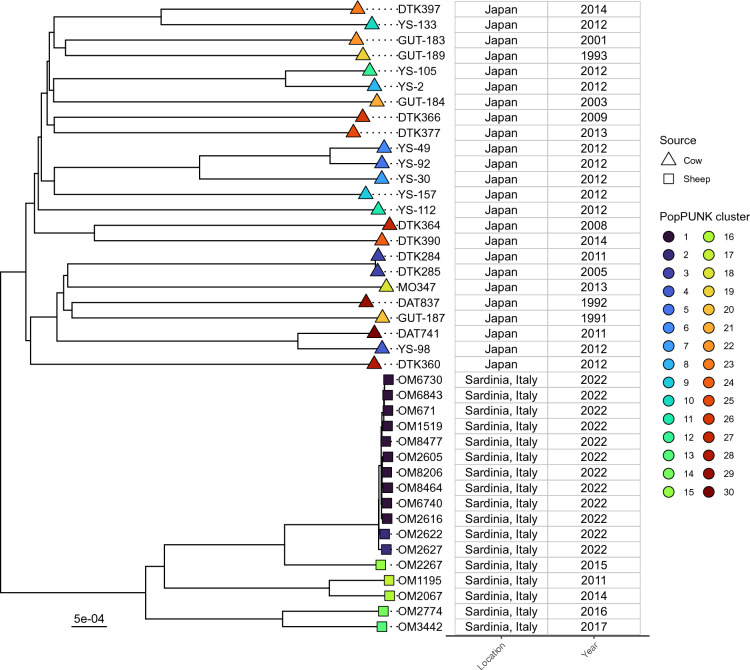
**Neighbour-joining tree of all publicly available *S. ruminantium* genomes.** Isolates coloured by PopPUNK cluster and source shown by tip shape.

The lineages of these isolates were then analysed in the context of all publicly available *S. ruminantium* genomes (Figure 2). The Sardinian sheep isolates isolated during this study clustered separately from the Japanese cow isolates and had a SNV mean of 7934 (range 7521–8226), indicating considerable divergence. Due the limited number of genomes currently available, it is unclear if this is a regional or host-specific observation. Notably, Japanese cow isolates were made up of distinct singleton lineages, except for two isolates (*S. ruminantium* DTK284 and DTK285) which were part of the same cluster (6 SNVs apart) despite being isolated 6 years apart. The phylogeny was also concordant with the SNV outbreak analysis regarding the 12 Sardinian isolates, where the SNV-linked outbreak (10 isolates) was clustered together with short branches, while also being part of the same PopPUNK cluster.

To determine any host-specific gene content, the pangenome of all *S. ruminantium* genomes was then analysed. A core genome of 1571 from of a total 4178 pan genes was identified (95% threshold), indicating a highly conserved gene set of 37.6% across all isolates. Forty-one genes were found to be specifically conserved in sheep isolates (Additional file 12). These mostly consisted of mobile genetic elements including transposes (4), integrases (2), phage proteins (7), as well as transcriptional regulator/DNA binding (8) and several metabolism and secondary metabolite-related genes (8). Three genes were found to be specifically conserved in cow-only isolates, consisting of *mdlB*, a ABC multidrug transport system and two hypothetical proteins.

### Virulence genes

**Figure 3:**
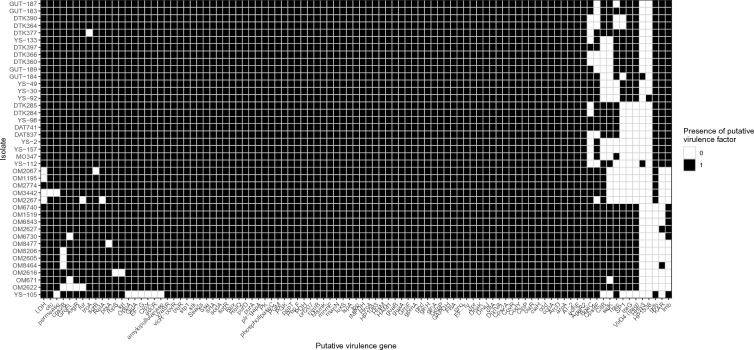
**Heatmap showing presence and absence of putative virulence factors in all *S. ruminantium* genomes used in this study.** Both axes were hierarchically clustered via Gower dissimilarity.

Putative virulence genes were screened using those identified in closely-related species *Streptococcus suis*. Most were core across all isolates (Figure 3 and Additional file 13) regardless of source of isolation, however there were a few notable exceptions. A 3-ketoacyl-ACP reductase (WP_032511883.1), laminin binding protein (WP_012774966.1) and glutamate dehydrogenase (WP_011921833.1) were found to be almost always present within cow isolates but absent in sheep mastitis isolates. Cow isolates were missing *citB* (CAZ51628.1) *cps9E* (AAF18948.1) and *bgaC*, a surface-anchored beta-galactosidase (WP_011922008.1).

## Discussion

These data provide the first global genomic insights into this multi-host ruminant pathogen. The presence of discrete, singleton lineages found to be the cause of pathogenesis in cows is consistent with the historical Sardinian sheep isolates in that these are likely to be opportunistic pathogens, as noted in other studies of *S. ruminantium* [11]. However, our study is the first to link *S. ruminantium* to a demonstrable outbreak. The presence of historical *S. ruminantium* isolates, initially identified as *S. suis* and collected between 2011–2017, indicates that this pathogenic species has been present in the region for over a decade. The clonal linkage of Japanese cow isolates *S. ruminantium* DTK284 and DTK285, isolated 6 years apart, shows that different clones can remain in circulation, but overwhelmingly these appear to be opportunistic infections caused by genetically distinct clones in both cattle and sheep. While mastitis is a serious clinical condition, there have been no *S. ruminantium*-associated endocarditis, respiratory disease, or death in sheep (Table 2), as has been reported in cows [4, 11, 22]. However, although comparisons were made between sheep and cow isolates in this study, they should be treated with caution due to the small number of genomes available. Given the severity of the disease in ruminants and the ability to spread clonally within a herd, surveillance efforts should be increased to monitor this important agricultural pathogen.

All Sardinian sheep isolates were resistant to the aminoglycoside tested, kanamycin, while being susceptible to all other classes tested (Additional file 2). This is broadly in agreement with a previous study of *S. uberis* mastitis in Sardinian sheep, which also showed high percentage of intrinsic resistance to aminoglycosides, but sensitivity to other antibiotics [19]. Notably, the historical isolates from 2016 onwards showed a similar antibiotic resistance profile to that of the 2022 outbreak. Characterization of these genetic resistance determinants is critical for future and informed surveillance of *S. ruminantium* and closely related pathogens. In contrast, many resistance genes were found in 16/24 Japanese cow isolates (median number of subclass resistances = 3). *tet(*M) or *tet*(O) tetracycline resistance was the most common (15 isolates), followed by *ant*(6)-Ia or *aad*E streptomycin resistance (12 isolates) then *erm*(B) macrolide resistance (10 isolates) (Additional file 11). The significant levels of antibiotic resistance, likely reflecting the use of these or related antimicrobials in that region [47].

At least 64 putative virulence genes were identified in *S. suis* that were present in each *S. ruminantium* genome in this study, further demonstrating their relatively recent divergence [4] and pathogenic overlap. Given that *S. suis* can also cause mastitis [48] and meningitis in cattle [49] and has previously been isolated by the Istituto Zooprofilattico Sperimentale della Sardegna during routine surveillance of sheep mastitis (unpublished data), it is likely that these pathogens share a similar opportunistic pathogenic strategy.

Future genomic sequencing of clinical isolates is recommended so that a greater number of genomes can be used to improve future genomic understanding of this under-represented but relevant agricultural pathogen. Short-term but frequent gastrointestinal colonisation and faecal shedding by the closely related *S. uberis* has been demonstrated [50] and it is possible that *S. ruminantium* follows a similar strategy. Therefore, we recommend molecular and/or genomic surveillance of non-clinical (healthy) host gastrointestinal tracts to determine non-clinical carriage rates, which would help to determine flock/herd susceptibility and allow early intervention to prevent disease onset, while flock outbreaks could be prevented by isolation of positive hosts.

### Supplementary Information

**Additional file 1: *Streptococcus *species included in the MBT Compass® Library Rev. K (2022).**

**Additional file 2: Isolates used in this study and their relevant information including MIC and MIC breakpoints.**

**Additional file 3: Protein fasta file of all putative virulence factors used in this analysis.**

**Additional file 4: All R code used to generate results in this study**

**Additional file 5: Genomic sequence of the *gap *gene and sequence similarity data for the *S. ruminantium* isolate n° 2622.**

**Additional file 6: Restriction fragment length polymorphism (RFLP) patterns of PCR products from the *gap* gene of 12 *S. ruminantium* and 2 *S. suis* isolates after digestion with *Alu*I enzyme and separated by 12% NuPAGE gel**. Lanes 1-12, isolates from mastitis outbreak; c1, *S. suis* isolate 3089; c2, *S. suis* isolate 3627. M, Marker VIII (Roche).

**Additional file 7: Agarose electrophoresis of amplicons. Panel A, PCR products (688 bp) from the *gdh* gene of twelve (lanes 1-12) *S. ruminantium* and two *S. suis* isolates (c1=3089; c2=3627).** Panel B, PCR products (336 bp) from the *rec*N gene using the same isolates of panel A. Panel C, PCR products (240 bp) from 16S rRNA gene using the same isolates of panel A. M, Marker VIII (Roche).

**Additional file 8: *Sma*I-digested PFGE patterns of *S. ruminantium* isolated from 12 sheep with mastitis belonging to the same flock.**

**Additional file 9: Output of pairwise SNV analysis as determined by SKA.**

**Additional file 10: Histogram showing the distribution of pairwise SNVs between all *S. ruminantium* genomes used in this study.**

**Additional file 11: Output of antimicrobial resistance analysis as determined by AMRFinder, presented in long table format.**

**Additional file 12: Output of Twilight of the pangenome analysis, based on isolate source.**

**Additional file 13: Summary of the putative virulence factors identified in two previous analyses from *Streptococcus suis.***
