# Supplementary material for: Streptococcus ruminantium-associated sheep mastitis outbreak detected in Italy is distinct from bovine isolates
Source: Vet Res. 2023 Dec 12;54:118. doi: 10.1186/s13567-023-01248-9 (PMC10717183; doi:10.1186/s13567-023-01248-9)
Supplement: Supplementary file 1 — Additional file 1: Streptococcus species included in the MBT Compass® Library Rev. K (2022). [file 13567_2023_1248_MOESM1_ESM.pdf]

Additional File 1 *Streptococcus* species included in the MBT Compass® Library Rev. K (2022), Document Revision E, covering 4274 species/entries.

Streptococcus acidominimus	*Streptococcus orisuis*
Streptococcus agalactiae	*Streptococcus ovis*
Streptococcus alactolyticus	*Streptococcus parasanguinis*
Streptococcus anginosus	*Streptococcus parauberis*
Streptococcus australis	*Streptococcus penaeicida*
Streptococcus caballi	*Streptococcus peroris*
Streptococcus canis	*Streptococcus phocae*
Streptococcus castoreus	*Streptococcus pluranimalium*
Streptococcus constellatus	*Streptococcus plurextorum*
Streptococcus criceti	*Streptococcus pneumoniae*
Streptococcus cristatus	*Streptococcus porci*
Streptococcus dentirousetti	*Streptococcus porcinus*
Streptococcus devriesei	*Streptococcus porcorum*
Streptococcus didelphis	*Streptococcus pseudopneumoniae*
Streptococcus downei	*Streptococcus pseudoporcinus*
Streptococcus dysgalactiae	*Streptococcus pyogenes*
Streptococcus entericus	*Streptococcus ratti*
Streptococcus equi_ssp_equi	*Streptococcus salivarius*
Streptococcus equi_ssp_zooepidemicus	*Streptococcus sanguinis*
Streptococcus equinus	*Streptococcus sinensis*
Streptococcus ferus	*Streptococcus sobrinus*
Streptococcus gallinaceus	*Streptococcus sp*
Streptococcus gallolyticus	*Streptococcus suis*
Streptococcus gordonii	*Streptococcus thermophilus*
Streptococcus halichoeri	*Streptococcus thoraltensis*
Streptococcus henryi	*Streptococcus uberis*
Streptococcus hyointestinalis	*Streptococcus urinalis*
Streptococcus hyovaginalis	*Streptococcus vestibularis*
Streptococcus infantarius	
Streptococcus infantis	
Streptococcus iniae	
Streptococcus intermedius	
Streptococcus lutetiensis	
Streptococcus macacae	
Streptococcus marimammalium	
Streptococcus massiliensis	
Streptococcus merionis	
Streptococcus minor	
Streptococcus mitis_oralis	
Streptococcus moroccensis	
Streptococcus mutans	
Streptococcus orisratti	
