# Supplementary material for: Streptococcus ruminantium-associated sheep mastitis outbreak detected in Italy is distinct from bovine isolates
Source: Vet Res. 2023 Dec 12;54:118. doi: 10.1186/s13567-023-01248-9 (PMC10717183; doi:10.1186/s13567-023-01248-9)

Additional file 5 Restriction fragment length polymorphism (RFLP) patterns of PCR products from the *gap* gene of 12 *S. ruminantium* and 2 *S. suis* isolates after digestion with *Alu*I enzyme and separated by 12% NuPAGE gel. Lanes 1-12, isolates from mastitis outbreak; c1, *S. suis* isolate 3089; c2, *S. suis* isolate 3627. M, Marker VIII (Roche)

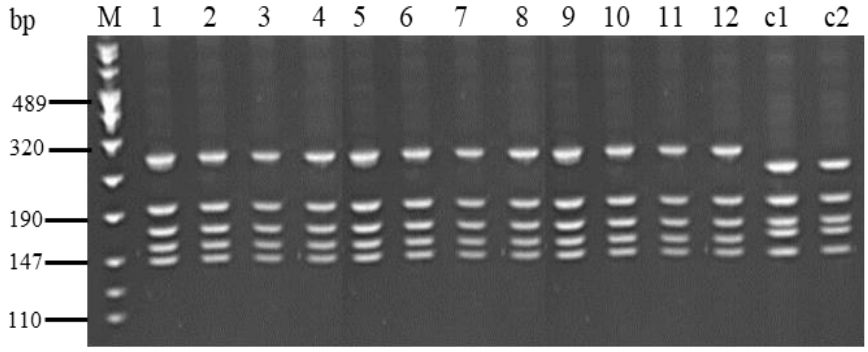

Supplement: Supplementary file 5 — Additional file 5: Genomic sequence of the gap gene and sequence similarity data for the S. ruminantium isolate n° 2622. [file 13567_2023_1248_MOESM5_ESM.pdf]
