# Supplementary material for: Streptococcus ruminantium-associated sheep mastitis outbreak detected in Italy is distinct from bovine isolates
Source: Vet Res. 2023 Dec 12;54:118. doi: 10.1186/s13567-023-01248-9 (PMC10717183; doi:10.1186/s13567-023-01248-9)
Supplement: Supplementary file 6 — Additional file 6: Restriction fragment length polymorphism (RFLP) patterns of PCR products from the gap gene of 12 S. ruminantium and 2 S. suis isolates after digestion with AluI enzyme and separated by 12% NuPAGE gel. Lanes 1-12, isolates from mastitis outbreak; c1, S. suis isolate 3089; c2, S. suis isolate 3627. M, Marker VIII (Roche). [file 13567_2023_1248_MOESM6_ESM.pdf]

Additional file 6 Genomic sequence of the *gap* gene and sequence similarity data for the S. *ruminantium* isolate n° 2622.

```
TCCGAATTGAGATACCCACGATGTTCTGAAGATACAAGTGGATCTTCAGTGTAACCGAATGATTCAGTAGCAGCAGTTTTCATAGCAGCGTTA
CTTCTTCAGCAGTTACTTTCTTATCAAGAGTCGCAACCAATTCAGTTACAGAACCTGTTGGAACTGGAACACGTTGTGCGGCACCATCAAGTT
TACCATTCAATTCTGGGATTACCAAACCGATAGCTTTAGCTGCACCAGTTGAGTTAGGAACGATGTTTGCTGCAGCAGCACGAGCACGACGAA
GGTCACCACCACGGTGTGGTCCGTCAAGAACCATTTGGTCACCAGTGTAACCGTGGATTGTAGTCATCAAACCTTTTTGAACGCCAAACGCAT
CGTGAAGAGCTTTTAGCCATTGGTGCCAAACAGTTTGTAGTACATGAAGCACCTGAGATAACTGTTTCAGTACCGTCAAGGATGTCATGGTTAG
TGTTGAAAACAACTGTCTTCACATCGTTACCACCAGGAGCAGTGATAACAACTTTTCTTAGCACCGTTAGCGTGAATGTGTTGCTCAGCTTTTT
CTTTTAGAAGCAAAGAAACCTGTTTGCTTCCAAAACAATATCTACGCCCATCAGTTAGCCCAGTTCAATGTTTTCCTGGGCTCACGCTCAGCAG
AAACTTTTAACGAATTTACCCGTTAACTTCAAAAACCACCCGTCTTTAACTTACAACCAGTACCCATCGAAAACGACCCTTTGA
```

select all *100 sequences selected* GenBank Graphics Distance tree of results MSA Viewer

	Description	Scientific Name	Max Score	Total Score	Query Cover	E value	Per. Ident	Acc. Len	Accession
☑	Streptococcus sp. DAT741 chromosome, complete genome	Streptococcus sp. DAT741	1212	1212	99%	0.0	96.99%	2105284	CP019557.1
☑	Streptococcus ruminantium GUT-189 DNA, complete genome	Streptococcus ruminantium	1212	1212	99%	0.0	96.99%	2081190	AP025333.1
☑	Streptococcus ruminantium GUT-183 DNA, complete genome	Streptococcus ruminantium	1212	1212	99%	0.0	96.99%	2175328	AP025331.1
☑	Streptococcus ruminantium GUT187T DNA, complete genome	Streptococcus ruminantium	1206	1206	99%	0.0	96.86%	2090539	AP018400.1
☑	Streptococcus ruminantium GUT-184 DNA, complete genome	Streptococcus ruminantium	1206	1206	99%	0.0	96.86%	2115310	AP025332.1
☑	Streptococcus suis strain NCTC10237 genome assembly, chromosome: 1	Streptococcus suis	1079	1079	99%	0.0	93.72%	2070644	LR594043.1
☑	Streptococcus suis strain 1081 chromosome, complete genome	Streptococcus suis	1079	1079	99%	0.0	93.72%	2228089	CP017667.1
☑	Streptococcus suis strain 0061 chromosome, complete genome	Streptococcus suis	1079	1079	99%	0.0	93.72%	2138420	CP017666.1
☑	Streptococcus suis ST1, complete genome	Streptococcus suis ST1	1079	1079	99%	0.0	93.72%	2034321	CP002651.1
☑	Streptococcus suis strain HN105 chromosome, complete genome	Streptococcus suis	1074	1074	99%	0.0	93.58%	2196724	CP029398.1
☑	Streptococcus suis strain M105052_S26 chromosome, complete genome	Streptococcus suis	1074	1074	99%	0.0	93.58%	2411696	CP102136.1
