# Supplementary material for: Streptococcus ruminantium-associated sheep mastitis outbreak detected in Italy is distinct from bovine isolates
Source: Vet Res. 2023 Dec 12;54:118. doi: 10.1186/s13567-023-01248-9 (PMC10717183; doi:10.1186/s13567-023-01248-9)

Additional file 7 Agarose electrophoresis of amplicons. Panel A, PCR products (688 bp) from the *gdh* gene of twelve (lanes 1-12) *S. ruminantium* and two *S. suis* isolates (c1=3089; c2=3627). Panel B, PCR products (336 bp) from the *rec*N gene using the same isolates of panel A. Panel C, PCR products (240 bp) from 16S rRNA gene using the same isolates of panel A. M, Marker VIII (Roche)

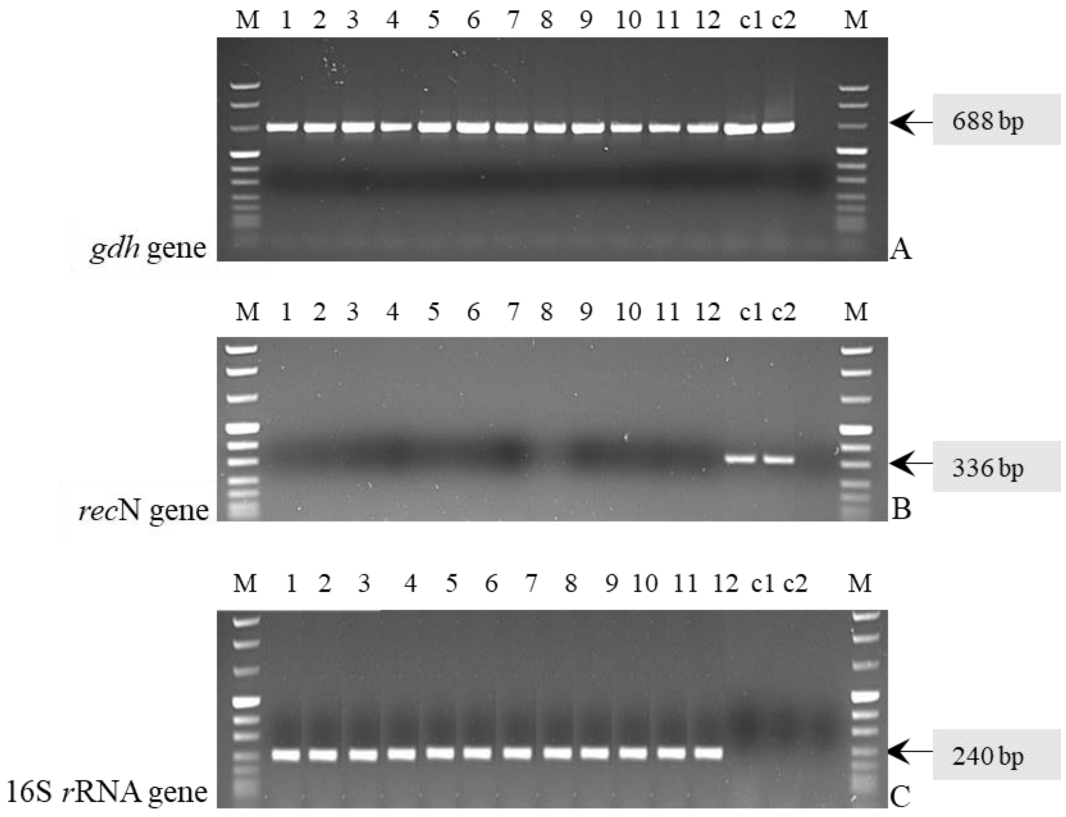

Supplement: Supplementary file 7 — Additional file 7: Agarose electrophoresis of amplicons. Panel A, PCR products (688 bp) from the gdh gene of twelve (lanes 1-12) S. ruminantium and two S. suis isolates (c1=3089; c2=3627). Panel B, PCR products (336 bp) from the recN gene using the same isolates of panel A. Panel C, PCR products (240 bp) from 16S rRNA gene using the same isolates of panel A. M, Marker VIII (Roche). [file 13567_2023_1248_MOESM7_ESM.pdf]
