# Supplementary material for: Streptococcus ruminantium-associated sheep mastitis outbreak detected in Italy is distinct from bovine isolates
Source: Vet Res. 2023 Dec 12;54:118. doi: 10.1186/s13567-023-01248-9 (PMC10717183; doi:10.1186/s13567-023-01248-9)

Additional file 8 *Sma*I-digested PFGE patterns of *S. ruminantium* isolated from 12 sheep with mastitis belonging to the same flock.

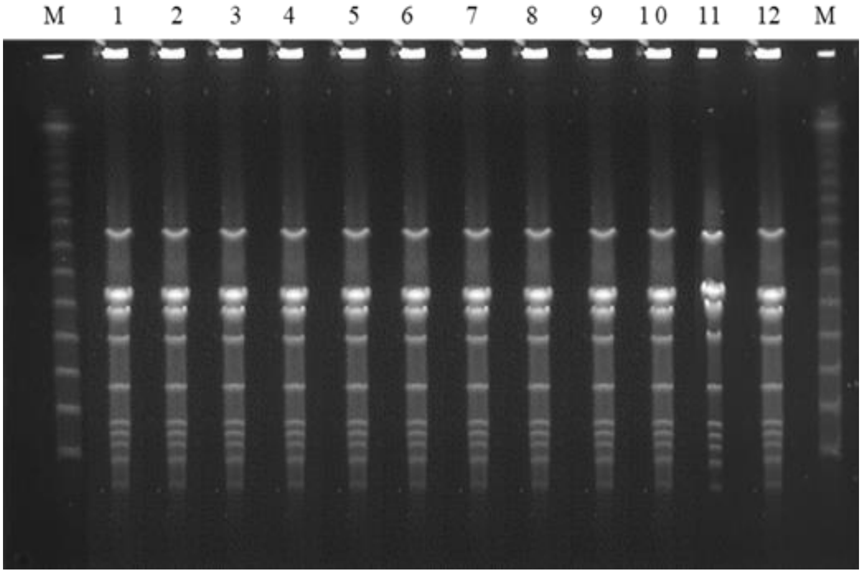

Supplement: Supplementary file 8 — Additional file 8: SmaI-digested PFGE patterns of S. ruminantium isolated from 12 sheep with mastitis belonging to the same flock. [file 13567_2023_1248_MOESM8_ESM.pdf]
