# Supplementary figures and images for: Streptococcus ruminantium-associated sheep mastitis outbreak detected in Italy is distinct from bovine isolates
Source: Vet Res. 2023 Dec 12;54:118. doi: 10.1186/s13567-023-01248-9 (PMC10717183; doi:10.1186/s13567-023-01248-9)

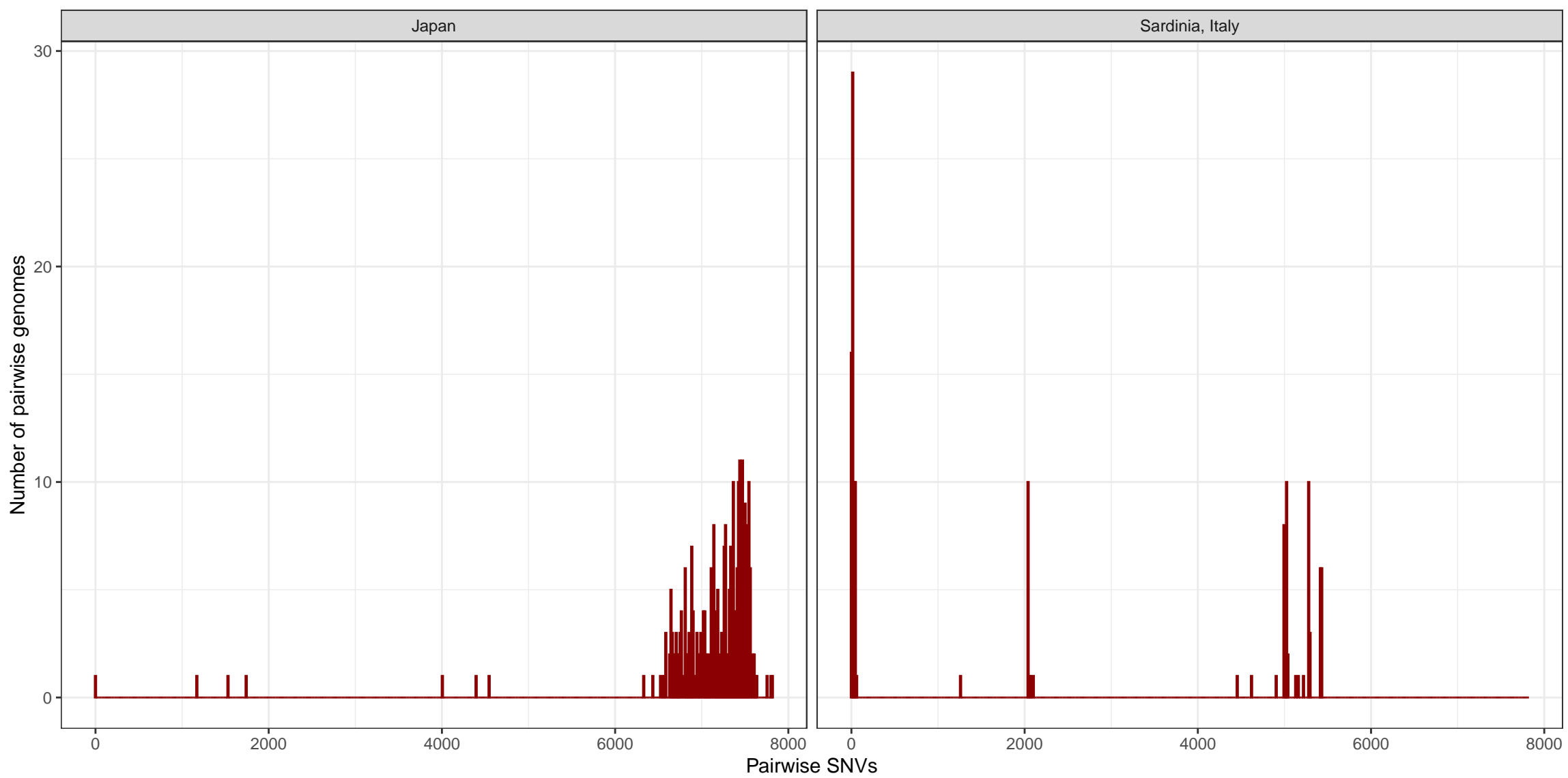

Supplement: Supplementary file 10 — Additional file 10: Histogram showing the distribution of pairwise SNVs between all S. ruminantium genomes used in this study. [file 13567_2023_1248_MOESM10_ESM.pdf]
